# Supplementary material for: Ultrasound-guided fine needle aspiration cytology and ultrasound examination of thyroid nodules in the UAE: A comparison
Source: PLoS One. 2021 Apr 7;16(4):e0247807. doi: 10.1371/journal.pone.0247807 (PMC8026079; doi:10.1371/journal.pone.0247807)
Supplement: Attachment — Submitted filename: Response to PLOS ONE reviewers comments.docx [file pone.0247807.s001.docx]

**Response to Reviewers comments**

**Reviewer #1**: In this paper, the authors intend to compare the accuracy of malignancy risk determination of thyroid nodules using US (ATA 2015 categorization), with cytology diagnosis (Bethesda system). This is an interesting and relevant subject.

Major issues:

1) Methods: How was the sample size determined?

**Response to reviewer’s comment**

Many thanks for your critical question. The sample size was determined by including all cases that have ultrasound examination of thyroid nodules and ultrasound-guided fine needle aspiration cytology that were followed by thyroid surgery and histopathological examination during the period 2011-2019 at Tawam Hospital in Al-Ain city, UAE. We have excluded patients who have thyroid nodules and underwent ultrasonography and FNA cytology examination but did not have thyroid surgery (lobectomy or thyroidectomy) with histopathologic diagnosis. Also, patients with incomplete data were not included in this study. Non-diagnostic category in Bethesda scoring system was also excluded from this study because the cases were not followed by surgery. All cases who have FNA or US reports of more than one year prior to the date of surgery were also excluded. All cases of thyroidectomy that have non-thyroid neoplasms were also excluded. We also exclude all cases who have thyroid surgery prior to FNA.

2) Methods: Why was the non-diagnostic Bethesda category excluded from the study?

**Response to reviewer’s comment**

Many thanks for your valuable comment. Non-diagnostic category (I) nodules in Bethesda scoring system were also excluded from this study because they were not followed by surgery, hence they did not have histopathological confirmation.

3) Methods: Data analysis mentions only the use of descriptive statistics, but the authors also performed an analytical study. The statistical tests used should be mentioned in this section.

**Response to reviewer’s comment**

Many thanks for your important question. All the used statistical methods were addressed in the data analysis section in the material and methods section.

“The data was extracted from the FNA cytology, US and histopathology reports of the patients who have undergone surgery during the study period. All data was analyzed using statistical package for the social sciences (SPSS) (version 20.0) using descriptive statistics. The chi-square test with Yates correction was used to compare between frequencies. Data are presented in mean ± standard error (S.E). P values < 0.05 are considered significant.

The accuracy test was done using the following formula:

Accuracy= True positive+True negative/ True positive+True negative+False positive+False negative

Histological diagnosis was taken as the gold standard and the FNA cytology and US diagnoses were compared to it.”

4) Methods/Results: Even though not clearly described in the methods section, in the results the authors present the comparison of the malignancy risk of each ultrasound category with a matched Bethesda category: Benign with Bethesda 2; very low suspicion with Bethesda 3; Low suspicion with Bethesda 4; intermediate suspicion with Bethesda 5, and High suspicion with Bethesda 6. Based on the results of this comparison, namely by comparing the malignancy risk of low suspicion nodules with the malignancy risk of Bethesda 4, they conclude that Cytology diagnosis is more accurate than US. In my opinion, this approach is inadequate to assess the research question.

**Response to reviewer’s comment**

Many thanks for your important comment. We have updated our results and made the comparison of risk of malignancy between the benign category of US and the benign category of FNA reports. In addition, we have made a comparison between the highly suspicious category of US with the malignant category of FNA reports which we think more relevant as we are comparing between two categories with almost well-defined features and nearly have similar weight.

5) Methods/results: The nodule’s malignancy risk was calculated based on a histopathology diagnosis of malignancy. However, the authors don’t explain how were the pathology specimens correlated with US images, and don´t mention the size range of the nodules submitted to FNA. How could the authors know for sure, in multinodular thyroids, which nodule had been submitted to US/FNA? This is particularly concerning due to the huge amount of microcarcinomas reported. They could be incidental findings, biasing the results.

**Response to reviewer’s comment**

Many thanks for your valuable comment. for this valuable and critical point. We have reviewed all the electronic files of both US and FNA reports. Following the review of nodules that had been examined by US and the nodules that have been examined by FNA and comparing the results with that of histopathological examination, we have identified that all microcarcinoma were incidental finding hence, they were removed from the calculation as malignancy.

6) Discussion, lines 361-362 – The statement “It is extremely difficult to diagnose follicular carcinoma by FNA” is incorrect, as it is not possible to diagnose follicular carcinoma by FNA.

**Response to reviewer’s comment**

Many thanks for your valuable comment. This sentence has been removed from the discussion.

7) Discussion, lines 395-396 – “BSRTC recommendation for management of patients in category 5 and category 6 is total thyroidectomy”. There is no reference to support this statement, which is actually inaccurate according to the recent update of the Bethesda System Reporting System.

**Response to reviewer’s comment**

Many thanks for your valuable comment. This sentence has been removed from the discussion.

8) There are many limitations in this study, not mentioned in the discussion section (examples: Retrospective, sample size, only one US and pathology reader, possible nodules that could not be included in any of the ATA categories, exclusion of nodules with Bethesda 1 diagnosis, pathology specimens’ correlation with the biopsied nodule…)

**Response to reviewer’s comment**

Many thanks for your critical comment. We have added a paragraph of limitations of this study at the end of discussion.

“We have limitations in our study, which may include sample size as well as being a retrospective study. However, studied material include all cases with thyroid nodules that fulfill the inclusion criteria in our tertiary care hospital during the period 2011-2019, besides, there is limited regional data comparing the risk of malignancy in thyroid nodules between FNA cytology and US examination. We have excluded TBRSTC category I from our study because they are non-diagnostic and the patients did not undergo thyroid surgery.”

9) Conclusion: the authors didn´t test the combination of US and FNA in predicting the malignancy of thyroid nodules. As such, the presented conclusion cannot be drawn from the results presented in this paper.

**Response to reviewer’s comment**

Many thanks for your valuable comment. We have modified the conclusion to become “In conclusion, FNA cytological examination is superior to ultrasonography in predicting malignancy in thyroid nodules. Thyroid nodules with the diagnosis of Bethesda category III&IV run high risk of malignancy thus more vigilance is warranted”. We have added a paragraph in the results measuring the accuracy of Bethesda category VI (malignant) and US ATA highly suspicious category. We have identified a higher accuracy of FNA reporting of Bethesda category VI (malignant) than US ATA reporting of highly suspicious category, which suggest that FNA cytological examination is superior to ultrasonography in predicting malignancy in thyroid nodules.

Minor Issues:

10) In the abstract methods subsection, the sample size is 161 nodules. However, the results section reports a total of 164 nodules.

**Response to reviewer’s comment**

Many thanks for your valuable comment.

The sample size is 161 and was corrected in the manuscript.

11) Even though the authors claim that US images were reviewed by a radiologist, there is no radiologist identified among the paper authors.

**Response to reviewer’s comment**

Many thanks for your valuable comment.

The 3^rd^ author is ^3^Dr. Maysam T Abu Sa'a, MD and she is a radiologist.

A second radiologist “Dr. Klaus Van Gorkom, is acknowledged in this manuscript for his participation in this study.

12) Bethesda 3 category is unusually named “atypical follicular cells of unknown significance” in table 2.

**Response to reviewer’s comment**

Many thanks for your valuable comment.

The Bethesda categories names were unified according to Bethesda system for reporting thyroid cytopathology in the tables and all manuscript data.

13) There are different abbreviations for the same words. Examples: FNA (line 177) and FNAC (line 185); US (line 176) and USS (line 269).

**Response to reviewer’s comment**

Many thanks for your valuable comment.

All abbreviations were unified in the manuscript.

14) There is a misplaced bullet in the bulleted list (line 254).

**Response to reviewer’s comment**

Many thanks for your valuable comment.

Misplaced bullet in the bulleted list is added.

15) References are inconsistent: example the journal name is in italic only in reference 4. There are different - _ separating the page numbers.

**Response to reviewer’s comment**

Many thanks for your valuable comment.

All references were reviewed and adjusted according to the journal requirement.

16) Language editing is recommended.

**Response to reviewer’s comment**

Many thanks for your valuable comment.

Language editing was done.

**Reviewer #2:** 164 surgically treated patients during 9 years means an extremely low number of cases. This low number of cases excludes the possibility to draw relevant conclusions from the data. Less than 20 histologically verified cases per year is insufficient to gain or maintain a high enough diagnostic standard for an everyday clinical practice and excludes the possibility that the
consequences drawn from this minimal practice could lead to any relevant and grounded

**Response to reviewer’s comment**

Many thanks for your valuable comment.

We agree with you that the sample size is low and it is one of the limitations of this study. We have addressed that in the discussion.

“We have limitations in our study, which may include sample size as well as being a retrospective study. However, studied material include all cases with thyroid nodules that fulfill the inclusion criteria in our tertiary care hospital during the period 2011-2019, besides, there is limited regional data comparing the risk of malignancy in thyroid nodules between FNA cytology and US examination as well as the accuracy in predicting malignancy in thyroid nodules in the UAE.”

In this study, large number of patients undergoing thyroid surgery for thyroid nodules were excluded for incomplete data set. As a tertiary care hospital, significant proportion of patients were partially investigated at the referring hospitals or centers.

22% malignancy rate among Bethesda 2 category is an extremely high false positive rate and
raises the possibility that the diagnostic standards used by the authors differ from the widely
applied criteria or might mean a failure of the evaluation process – either the aspiration
technique could be inappropriate or the cytopathologist might had only limited practice with
thyroid FNA smears.

**Response to reviewer’s comment**

Many thanks for your valuable and critical comment.

We have thoroughly reviewed all the electronic files of US, FNA reports and corresponding histopathological reports. Following the review of nodules that had been examined by US and the nodules that have been examined by FNA and comparing the results with that of histopathological examination we have identified that all microcarcinoma were incidental finding and were not within the sampled nodules by FNA , and they were removed from the calculation as malignancy, hence the risk of malignancy for Bethesda category II (benign) becomes 3% which is within the range of risk of malignancy reported by Bethesda system for reporting thyroid cytopathology (TBSRTC) which is 0-3%.
